# mtDNA Heteroplasmy at the Core of Aging-Associated Heart Failure. An Integrative View of OXPHOS and Mitochondrial Life Cycle in Cardiac Mitochondrial Physiology

**DOI:** 10.3389/fcell.2021.625020

**Published:** 2021-02-22

**Authors:** Alvaro A. Elorza, Juan Pablo Soffia

**Affiliations:** ^1^Faculty of Medicine and Faculty of Life Sciences, Institute of Biomedical Sciences, Universidad Andres Bello, Santiago, Chile; ^2^Millennium Institute on Immunology and Immunotherapy, Santiago, Chile

**Keywords:** cardiac, OXPHOS, ROS, mtDNA heteroplasmy, aging, heart failure, mitophagy, biogenesis

## Abstract

The most common aging-associated diseases are cardiovascular diseases which affect 40% of elderly people. Elderly people are prone to suffer aging-associated diseases which are not only related to health and medical cost but also to labor, household productivity and mortality cost. Aging is becoming a world problem and it is estimated that 21.8% of global population will be older than 65 years old in 2050; and for the first time in human history, there will be more elderly people than children. It is well accepted that the origin of aging-associated cardiovascular diseases is mitochondrial dysfunction. Mitochondria have their own genome (mtDNA) that is circular, double-stranded, and 16,569 bp long in humans. There are between 500 to 6000 mtDNA copies per cell which are tissue-specific. As a by-product of ATP production, reactive oxygen species (ROS) are generated which damage proteins, lipids, and mtDNA. ROS-mutated mtDNA co-existing with wild type mtDNA is called mtDNA heteroplasmy. The progressive increase in mtDNA heteroplasmy causes progressive mitochondrial dysfunction leading to a loss in their bioenergetic capacity, disruption in the balance of mitochondrial fusion and fission events (mitochondrial dynamics, MtDy) and decreased mitophagy. This failure in mitochondrial physiology leads to the accumulation of depolarized and ROS-generating mitochondria. Thus, besides attenuated ATP production, dysfunctional mitochondria interfere with proper cellular metabolism and signaling pathways in cardiac cells, contributing to the development of aging-associated cardiovascular diseases. In this context, there is a growing interest to enhance mitochondrial function by decreasing mtDNA heteroplasmy. Reduction in mtDNA heteroplasmy is associated with increased mitophagy, proper MtDy balance and mitochondrial biogenesis; and those processes can delay the onset or progression of cardiovascular diseases. This has led to the development of mitochondrial therapies based on the application of nutritional, pharmacological and genetic treatments. Those seeking to have a positive impact on mtDNA integrity, mitochondrial biogenesis, dynamics and mitophagy in old and sick hearts. This review covers the current knowledge of mitochondrial physiopathology in aging, how disruption of OXPHOS or mitochondrial life cycle alter mtDNA and cardiac cell function; and novel mitochondrial therapies to protect and rescue our heart from cardiovascular diseases.

## Introduction

The world is aging at a very high speed and with it, the aging-associated diseases are going up. According to Lutz et al. (2008), the proportion of the global population of 60 + years was 10% in 2000 but is projected to be 21.8% in 2050 and then to 32.2% in 2100. Current projections on aging show that in 2050 there will be more elderly people (65+) than children (0-14 years old) for the first time in human history. This is enormous pressure for the health care system and for the economy of any country because older adults are affected by several and diverse chronic diseases - aging-associated diseases- that contribute to disability, diminish the quality of life and increase health- and long term- care costs. The cost of aging-associated diseases is not only related to medical care but also to labor and household productivity losses and mortality costs. Furthermore, old people in many cases are affected by a combination of diseases which increases the cost even more. Older people are more susceptible to get ill when they work or live under environmental risk factors such as air pollution, tobacco smoke, particulate matter and ozone and/or under low quality of life risk factors such as sedentarism and bad food quality, leading to obesity. This negative environmental exposure may cause or exacerbate respiratory and cardiovascular diseases (Van Houtven et al., 2008). Cardiovascular diseases are the leading cause of death among older people reaching up to 40% of deaths in people over 65 years old (Van Houtven et al., 2008; Ginneken, 2017). Therefore, new heart failure treatments are of high relevance to improve the life quality and lifespan of older people and also to decrease the pressure and costs on the public health system. Working on new methodologies and technologies will make a contribution to expediting and advancing drug discovery and genetic therapies in this area.

Our heart is a wonderful organ built at the beginning of our existence, during embryonic development, and never ever stops working until we die. It is a perfect machine that pumps, day and night, blood into all tissues, resting only between beats. Furthermore, if we exercise or have an increase in oxygen demand, our heart beats even harder to satisfy tissue demands. It has been estimated that our heart beats 36 million times per year and to support such a high load of work, our heart must be able to produce an enormous amount of ATP that has been estimated in about 30-40 Kg per day (Ferrari et al., 2003) which is about one-third of total ATP produced by our body (a normal individual produces 90-100 Kg of ATP daily). Cardiac cells are contractile striated cells with an average render cell volume of 30.5 pL (picoLiters) and 140 μm × 32 μm × 13 μm (LxWxD) average dimension in mammals. The cardiac cell capacitance, which is proportional to the amount of membrane and membrane invaginations, varies among different mammal species between 138 and 300 pF (picoFaraday). The amount of membrane invaginations is explained by the presence of T-tubules (50% membrane), endoplasmic reticulum and mitochondria which are all involved in cardiac cell contraction (Satoh et al., 1996). The ultrastructural analysis of cardiac cells also reveals that the volume density of mitochondria is over 25.3%, the myofibrils 52.3% and the cytoplasm 22.3%. One-third of the cardiac volume is filled with mitochondria (Barth et al., 1992); although Huang et al. (2013) estimate mitochondrial volume to be up to 40%.

## Keeping Healthy and Happy Mitochondria: OXPHOS and MLC

Mitochondria are cellular organelles that are well known as the powerhouse of the cell in charge of satisfying ATP demands. However, today, mitochondria are also acknowledged as a metabolic hub that integrates intracellular signaling to elaborate and execute a cellular response to adapt cell metabolism to external or internal environmental changes, taking primary action in cell fate determination. Fit and healthy mitochondria will be able to satisfy the heart’s energy demands, but dysfunctional mitochondria won’t, therefore a cardiac failure is expected.

**FIGURE 1:**
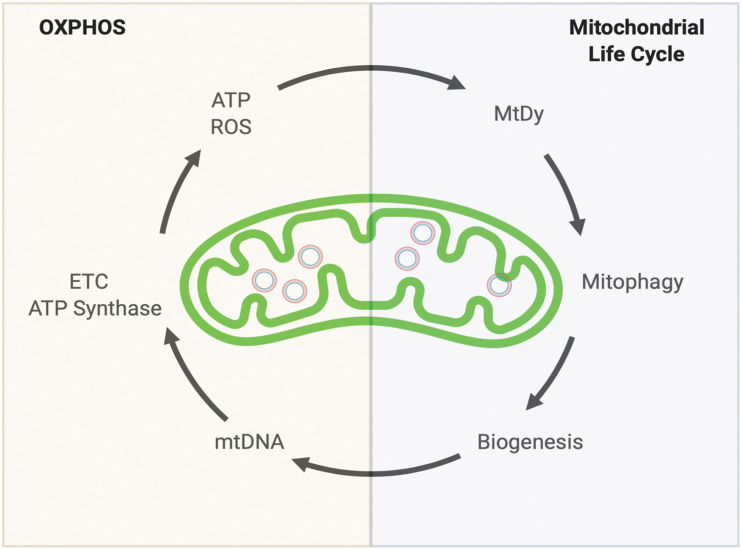
Keeping a healthy mitochondrial population. Mitochondria’s health depends on two complementary systems. The first system is the OXPHOS. This will provide energy (ATP) and reactive oxygen species (ROS) which are both necessary to drive the second system: The Mitochondrial Life cycle. Through mitochondrial fusion and fission events (Mitochondrial dynamics, MtDy), mitochondria are segregated to be eliminated by mitophagy. The induction of mitophagy is coupled with mitochondrial biogenesis which involves mitochondrial DNA (mtDNA) replication. mtDNA encodes for 11 respiratory complex subunits (7 subunits for complex I, 1 for complex III, 3 for complex IV) plus 2 subunits of the ATP synthase that will allow OXPHOS assembly to keep going the virtuous circle. Any disruption or delay in this running circle will end up in the loss of energy, mutations in mtDNA, decrease in mtDNA copy number and increased ROS leading to mitochondrial dysfunction, cell death, aging and aging-associated diseases.

In general, for all of our cells (excluding erythrocytes that do not have mitochondria) two convoluted mitochondrial systems work collaboratively to maintain a healthy mitochondrial population. The Oxidative Phosphorylation (OXPHOS) system and the Mitochondrial Quality Control System also known as the Mitochondrial Life Cycle (MLC). Together they complement each other to keep mitochondrial function i.e., energy production (ATP), reactive oxygen species (ROS) detoxification, proper metabolism and retrograde signaling (Figure 1).

OXPHOS is made of the Electron Transport Chain (ETC) to generate the proton motive force (mitochondrial membrane potential) and the ATP synthase that utilizes the proton motive force to generate ATP. The ETC or respiratory chain is formed by 4 respiratory complexes plus the ubiquinone and the cytochrome c, located in the inner mitochondrial membrane that forms cristae. OXPHOS is fed by NADH at the level of complex I and FADH2 at the level of complex II. Both reductant equivalents are mostly generated by the Krebs (TCA) cycle which in turn is dependent on calcium and respiratory substrates such as carbohydrates and lipids. As a byproduct of the respiratory chain, the superoxide radical (a ROS molecule) is generated, which has been involved in aging and aging-associated diseases. In this way, a very simplistic OXPHOS equation is:

Respiratory⁢Substrates+Calcium=NADH+FADH2=ATP+ROS+Heat

The OXPHOS system is encoded by two independent genetic material: nuclear DNA and mitochondrial DNA (mtDNA). The mtDNA is a plasmid DNA of 16 kb (circular, closed), doubled stranded and codes for 37 genes (13 OXPHOS protein-encoding mRNA, 2 rRNA and 22 tRNA). It is composed of a heavy strand and a light strand based on the proportion of heavier nucleotides (adenine and guanine). It also has a large non-coding region (around 1 kb) that contains the regulatory elements for the initiation and termination of the transcription of both strands. The D-Loop (Displacement Loop) or control region, is within this non-coding zone and contains the origin of replication of the heavy strand and high-variability zones called HVRI, HVR II and HVR III where most of the polymorphisms associated with mtDNA occur. Each molecule of mtDNA is packaged in structures called nucleoids made of proteins involved in their replication and transcription, such as TFAM. Nucleoids are located in the mitochondrial matrix associated with the internal membrane and the oxidative phosphorylation system (Brown et al., 2011).

Mitochondria have their own genome is maternally inherited and this configures each individual with their own maternal surname - called a haplogroup. All mitochondrial haplogroups derive from a common ancestor, from a black African woman, called “The Mitochondrial Eve” from 200,000 years ago (Cann et al., 1987). At present, a total of 20 haplogroups are recognized, each with its respective subdivisions, which were established by the acquisition of mutations and natural selection, according to the patterns of migration and human settlement, and fixed in each of the different ethnic groups. This is how the haplogroup J, D4a, D4b2b, D5 have been associated with longevity; the H5 and United Kingdom, at risk of suffering from Alzheimer’s disease; the U, with a predisposition to psychosis in bipolar disorders; and others associated with resistance to altitude, with athletic performance, predisposition to diabetes, cancer or cardiovascular diseases (Hiona, 2008; Lakatos et al., 2010; Nishigaki et al., 2010; Picard et al., 2016; Chocron et al., 2019). Knowing that the haplogroup confers adaptive advantages and/or disease propensity and, therefore, their identification can also become a tool of preventive diagnosis that can be applied since the birth of a new being.

mtDNA is prone to oxidative or replicative damage. Each mitochondrion has 3 to 10 mtDNA copies which undergo mutations by either the reactive oxygen species (ROS) generated as a byproduct of electron transfer chain due to electron leak mainly from respiratory complex I and III (Brand et al., 2013) or alterations in the mtDNA replication or repair. A higher mutation load in mtDNA means lower OXPHOS functionality giving rise to more ROS and more mtDNA mutations in a vicious cycle. The coexistence of wild type mtDNA and mutated mtDNA in the same mitochondrion is known as heteroplasmy. As the percentage of mutant mtDNA rises above certain thresholds (60-70%), cellular homeostatic mechanisms are disrupted, leading to mitochondrial dysfunction, cellular atrophy, and death, contributing to age-related diseases. Heteroplasmy also leads to broad changes in gene expression that can shift abruptly as the percentage of mutant mtDNA increases (Lakshmanan et al., 2018). To date, more than 400 mtDNA mutations have been associated with human diseases (Li et al., 2010).

The mitochondrial life cycle involves three processes: mitochondrial dynamics, mitochondrial selective autophagy (mitophagy) and mitochondrial biogenesis. Through MtDy, mitochondria share or dilute components and also segregate dysfunctional mitochondrial units for degradation by mitophagy (Legros et al., 2002; Liesa et al., 2009). While mitochondria are being degraded, mitochondrial biogenesis is turned on to maintain mitochondrial population and homeostasis (Palikaras et al., 2015a; Sin et al., 2016). In addition, mitochondrial biogenesis responds to energy demands and environmental factors such as cold and hypoxia (Tohme et al., 2017; Gureev, 2019).

Mitochondrial Dynamics (MtDy) refers to the ability of two mitochondria to fuse each other (fusion) or one mitochondrion to divide into two daughter mitochondria (fission). Mitochondrial morphology and size are dependent on mitochondrial fusion and fission events. Importantly, changes in MtDy regulate bioenergetics outputs including respiratory rate, energy expenditure and ATP synthesis as well as apoptosis and the segregation and elimination of dysfunctional mitochondrial units by mitophagy (Bénard et al., 2007; Sheridan and Martin, 2010; Westermann, 2012). Mitochondrial fission is dependent on the protein DRP1 that has been recognized as a mitochondrial fission promoter (Smirnova et al., 2001). It is located in the cytosol, but translocate to mitochondria through its binding to the mitochondrial receptor proteins FIS1, MFF, MiD49, and MiD51 (Losón et al., 2013), which are all located in the mitochondrial outer membrane (MOM). Mitochondrial fusion is dependent on MFN1 and MFN2, located in the MOM, and OPA1, which is located in the mitochondrial inner membrane (Liesa et al., 2009). A fusion event is often followed by a fission event generating one hyperpolarized and one depolarized daughter mitochondrion. The depolarized unit loses OPA1, MFN1, and MFN2 and thus its ability to fuse, becoming a target for degradation by mitophagy; while the hyperpolarized unit remains in the cytosol and can fuse again. In this way, cells keep a more active, healthier and functional mitochondrial web (Twig et al., 2008).

Mitochondrial Selective Autophagy or Mitophagy is a type of macroautophagy that delivers mitochondria to lysosomes for degradation. It is essential for the recycling and protective process of mitochondria to maintain cellular homeostasis. Mitophagy initiates with the formation of a double-membrane phagophore which elongates and closes to generate a mature, double-membrane autophagosome that engulfs mitochondria. Then, autophagosomes are fused to lysosomes for content degradation (Youle and Narendra, 2011). Mitophagy allows the removal of dysfunctional, depolarized and/or damaged mitochondria for mitochondrial turnover, ROS management, and programmed mitochondrial clearance, as seen in erythropoiesis (Schweers et al., 2007), lens differentiation (Bassnett, 2002), and mature T-lymphocytes (Pua et al., 2009). Mitophagy is also essential for embryonic development by removal of paternal sperm mitochondria from the fertilized eggs, leaving only the maternal ones (Rojansky et al., 2016; Pickles et al., 2018). In this regard, cells display basal mitophagy, stress-induced mitophagy and programmed mitophagy, being the cardiac muscle one of the tissues with enhanced basal mitophagy (Palikaras et al., 2018).

The process of mitophagy requires two systems: The autophagic core machinery, which is common to all types of autophagy; and the mitochondrial receptors and adaptors, a specific set of mitochondrial and/or cytosolic proteins needed for the assembly of mitochondria with the autophagic core machinery. The Autophagic Core Machinery is composed of ATG proteins that are found from yeast to mammals, indicating that autophagy is an evolutionarily conserved process. Phagophore initiation and formation is dependent on the ATG16L1 complex, made of ATG16L1, ATG5, and ATG12 which localize to the isolation membrane and dissociates from it upon the completion of autophagosome formation; and ATG8/LC3, which upon the formation of autophagosome, remains in on both sides—inside and outside of the double-membrane structure. While in yeast there is a single ATG8 protein, in mammals there are seven orthologs of ATG8 named LC3A, LC3B, LC3C, GABARAP, GABARAPL1, GABARAPL2, GABARAPL3 (thereafter they will be called LC3) suggesting a complex diversification of their function. LC3/ATG8 can be found in two isoforms: LC3-I and LC3-II. LC3-I is the inactive form that is constitutively expressed and found in the cytosol. Upon induction of autophagy, LC3-I is converted to LC3-II by site-specific proteolysis and lipidation near to the C-terminus to generate the autophagosome. LC3 is considered a reliable marker for on-going autophagy (Ashrafi and Schwarz, 2013; Lee and Lee, 2016).

The Mitochondrial Receptors interact directly or indirectly through adaptors, with LC3 via the LC3-interacting Region (LIR), a tetrapeptide sequence W/YXXL/I. Independent and non-redundant mitophagy pathways have been defined according to the mitochondrial receptors and adaptors that are involved in it. Those are (i) Ubiquitin-Dependent Mitophagy, with the PINK1-PARKIN protein axis. PINK1 (PTEN-induced putative protein kinase 1) is a mitochondrial protein whose import is dependent on mitochondrial membrane potential. Once imported, PINK1 is degraded by mitochondrial proteases. However, if mitochondria become depolarized, PINK1 is no longer imported and accumulates on the mitochondrial surface to phosphorylate ubiquitin and Mfn2, allowing the recruitment of the E3 ubiquitin ligase PARKIN from the cytosol. PARKIN mediates a hyper-ubiquitination of the mitochondrial outer membrane proteins, which are recognized by ubiquitin-binding adaptors such as P62/SQSTM1, OPTN, TAX1BP1, NDP52, NBR1, TOLLIP and HDAC6. These adaptor proteins interact and bind LC3 by means of their LIR domain. Of note, P62/SQSTM1 has been also used as a reliable indicator of the mitophagic flux, because under mitophagic defects it accumulates in response to stress stimuli (Xu et al., 2015; Yamaguchi et al., 2016). This PINK/PARKIN mitophagy is the canonical pathway for mitochondrial turnover and mitochondrial quality control. (ii) Ubiquitin-Independent Mitophagy is dependent on mitochondrial receptors interacting directly with LC3 through the LIR domain. Several and diverse mitochondrial LC3 receptors have been discovered, which also account for the complexity of mitophagy. NIX, BNIP3 (Zhang and Ney, 2009), FUNDC (Liu et al., 2012), FKBP8 (Bhujabal et al., 2017; Yoo et al., 2020), NLRX1 (Zhang et al., 2019) are located in the MOM; Prohibitins (Wei et al., 2017), in the MIM and, MsrB2 in the mitochondrial matrix (Lee et al., 2019). Not only proteins may bind LC3, but also the membrane phospholipid cardiolipin (Chu et al., 2013) mainly found in the MIM. NIX protein is highly expressed in erythroid precursors and is needed for erythropoiesis (Novak et al., 2009). Also, NIX, BNIP3 and FUNDC trigger mitophagy in response to hypoxia (Sowter et al., 2001; Bellot et al., 2009; Liu et al., 2012), Prohibitins, MsrB2 and cardiolipin could be accessible to cytosolic proteins if MOM or MIM are disrupted.

Mitochondrial dynamics and the mitophagy pathways with their mitochondrial receptors, adaptors and regulator proteins that localize to mitochondria, seem to have not redundant functions and might act cooperatively, providing multiple mechanisms to clear out damaged mitochondria under different conditions. In fact, the mitophagy protein FUNDC1 has been shown to interact with the MtDy proteins DRP1 or OPA1 to coordinate mitochondrial fission or fusion and mitophagy. Upon mitochondrial stress, FUNDC1-OPA1 interaction is dismissed, promoting DRP1 translocation to mitochondria for fission and a new association FUNDC1-DRP1 for mitophagy. This reveals the complexity and importance that MtDy and Mitophagy play for cellular physiology.

Mitochondrial Biogenesis: When mitochondria are eliminated by mitophagy or when more mitochondria are needed to meet energy demands, the mitochondrial biogenesis program is started. Interestingly, mitochondrial biogenesis involves four independent processes: mtDNA replication, mtDNA transcription, protein translation and mitochondrial membrane biogenesis, which are not necessarily synchronized adding more complexity and regulation capabilities to mitochondria. Unfortunately, the mechanisms controlling these three processes are not fully understood or discovered. In fish acclimated to cold, it was shown that mitochondria are able to remodel differentially according to ATP demands, oxygen demands, or both. Under oxygen demands, membrane biogenesis is upregulated increasing the mitochondrial volume/mass but not OXPHOS protein densities. Having an extra membrane improves oxygen diffusion since oxygen diffuses easily and faster in lipidic than in an aqueous environment. Under ATP demand, mitochondria increase the OXPHOS densities without increasing their volume. In the case of ATP and oxygen demands, both protein density and membrane are increased (O’Brien, 2010). On the other hand, it has been reported that mtDNA replication is controlled by oxidative damage in the D-loop region which favors TFAM (a mitochondrial transcription factor) binding to mtDNA for replication. This oxidative damage may be caused by hypoxia, linking the lack of oxygen to mtDNA replication (Pastukh et al., 2016).

## Mitochondria in Cardiac Cells

The aforementioned mitochondrial features for OXPHOS and mitochondrial life cycle are general for all mitochondria. But what is distinctive for heart mitochondria? (Table 1). Heart striated cells are special and different from other cell types. They have a particular shape and contract throughout our lives from early development until we die. Heart cells demand an enormous amount of energy and then mitochondria must do the work. Mitochondria in cardiac cells have been subdivided into interfibrillar, subsarcolemmal and perinuclear mitochondria, according to their distribution in the cell; and are tightly packed between myofibrils with almost no chance of free or flexible movement. However, Hendgen-Cotta et al. (2018) claimed that all cardiac mitochondria in mice are interconnected and, therefore, they are not different populations. Cardiac mitochondria seen under transmission electron microscopy look well organized to each other and actually mitochondrial cristae seem to be aligned between two adjacent mitochondria. Three-dimensional reconstruction of murine heart mitochondria has shown that they have an oval but irregular shape. Changes in mitochondrial morphology have been associated with metabolic and bioenergetic reprogramming. The heart is not the exception since most of the cardiopathies are associated with alteration in mitochondrial morphology. Fragmented, swollen, reduced mitochondrial number, reduced cristae densities are the main morphological features of human heart failure which are nicely tabulated in Daghistani et al. (2018). Interestingly, different cardiac pathologies have shown distinct morphological mitochondria and cristae patterns. Heart mitochondrial cristae are very long, mostly going all throughout the mitochondrial matrix. In comparison, cristae length in non-cardiac mitochondria is about 1/3-1/4 of mitochondrial width. Cristae hold the OXPHOS system and considering the length of cristae and the electrodense nature of mitochondrial matrix seen by TEM (in accordance with condense mitochondrial state and opposed to orthodox one), it is possible to suggest that cardiac mitochondria are fully packed with OXPHOS having a highly oxidative metabolism. In fact, nice work from Williams et al. (2018) analyzed the mitochondrial proteome from 5 different mouse tissues and showed that in heart tissues half of the total proteome (55%) correspond to mitochondrial proteins, which was higher than brain, liver, skeletal muscle and brown adipose tissue. When looking at OXPHOS protein expression, cardiac mitochondria displayed the highest OXPHOS protein density for both all ETC respiratory complexes and the ATP synthase as compared with other tissues.

**TABLE 1:** Summary of main characteristics found in mitochondria from non-cardiac cells, cardiomyocytes and aged cardiomyocytes.

	Young Standard Mitochondria*	**Young Cardiac Mitochondria**	**Aged-Cardiac Mitochondria**
**mtDNA**			
Mutation levels	– Low	– Low	– High
Heteroplasmy levels	– Low (<60%)	– Low (<60%)	– High (>60%)
Copy number	– High	– High	– Low
**Biomass and Morphology**			
Cell Occupancy (% of total cell Volume)	3-8%	30-40%	<30-40%
Subpopulations	– None	– Interfibrillar, subsarcolemmal and perinuclear	– Interfibrillar, subsarcolemmal and perinuclear
Mitochondrial Network	– Mostly tubular shape and branched mitochondria	– Mostly oval with irregular shape. – Highly packed between myofibrils.	– Fragmented, Shrunken or Swollen
Cristae Dimensions	– Length: 1/3 of mitochondrial width. (12 to 40 nm)	– Length: All throughout mitochondrial width (30-40 nm) – Aligned between adjacent mitochondria	– Altered cristae structure.
Morphological state seen under TEM	– Orthodox state	– Condensed state	– Orthodox, Condense and Swollen states
**Bioenergetics**			
OXPHOS protein Density	– Middle	– High	– Middle-Low
Special OXPHOS subunit isoforms	– Most tissues have ubiquitous COX subunit isoform expression	– Special Heart (H) Subunit Isoforms COX VIa H COX VIIa H COX VIII H	– Unknown
OXPHOS post-translational modifications	– Low levels of O-GlcNAcylated proteins	– High levels of O-GlcNAcylated proteins	– Unknown
Main Respiratory Substrate	– Glucose	– Fatty Acids – Triacylglycerides	– Glucose
ATP production	20-50%	80%	<80%
Calcium management	100nM	100-800nM	– Disrupted Calcium Homeostasis
ROS production	– Low	– Low-Middle	– High
mPTP status	– Physiological Flickering	– Desensitized to deal with increased Ca^++^ uptake and ROS	– Sensitized. Pathological Flickering and Opening
Mitoflashes	– Low (mPTP dependent?)	– Low (mPTP dependent?)	– High (ANT dependent)
**Mitochondrial Life Cycle**			
Predominant control of mitochondrial biomass	– PGC-1α – NFE2L2 (Activators of Mito Biogenesis)	– PGC-1α – NFE2L2 (Activators of Mito Biogenesis)	– NcoRI – SMRT? – RIP140? (Repressors of Mito Biogenesis)
Mitochondrial fusion and fission events	– Frequent events between adjacent mitochondria	– Frequent events between adjacent mitochondria. – Nanotunnel generation for long distance fusion events	– Decreased fusion and fission events. – Decreased nanotunnel generation?
Predominant basal mitophagy pathway	– PINK/PARKIN/P62	– PINK/PARKIN/P62. – Autophagy-dependent exospheres release	– Decreased PINK/PARKIN/P62 pathway. – Low Autophagy-dependent exospheres release. – Increased receptor mediated mitophagy gene expression. – Increased MUL1 expression?

* General features found in mitochondria from different types of cells. Variations may exist.

***?** Expected to occur but not demonstrated yet. TEM, Transmission Electron Microscopy.*

The super oxidative metabolism of cardiac cells is explained by the use of triacylglycerols and fatty acids as the main oxidative fuels, followed by glucose and other carbohydrates, which enter to the TCA cycle-OXPHOS to produce 80-90% of total ATP. The relative contribution of fat and carbohydrate to energy provision for the heart is 70% and 30%, respectively (Stoll et al., 2018) (Taegtmeyer et al., 2016). Furthermore, OXPHOS in cardiac mitochondria is equipped with specific subunit isoforms for the cytochrome c oxidase (complex IV) to make the ETC more efficient (Sinkler et al., 2017). The assembly of respiratory complexes in supercomplexes or respirosomes, reported for bovine, ovine and porcine hearts (Letts et al., 2016; Sousa et al., 2016) plus post-translational modifications in respiratory complexes such as O-GlcNAcylation, the ability to increase calcium uptake, and de-sensitize the opening of the mPTP can also improve OXPHOS efficiency (Ma et al., 2015; Stoll et al., 2018). OXPHOS efficiency is not exclusively dependent on OXPHOS proteins but also on other proteins involved in the bioenergetic capacity of cardiac cells. This is the case of the Adenine Nucleotide Transporter (ANT) protein, which is nuclear-encoded and allows the exchange of ATP by ADP through the inner mitochondrial membrane. ANT KO mouse display impaired activity of complex I, increase in ROS and oxidative damage. They also display a reduction in OPA1 leading to a fragmented mitochondrial web and sensitization to the opening of mPTP which has been associated with cardiopathies (Strauss et al., 2013).

Heart mitochondria have an active mitochondrial life cycle. Albeit to be highly packed with almost no freedom for movement, mitochondrial dynamics, mitophagy and biogenesis are critical for heart function (Eisner et al., 2017; Wu et al., 2019). Heart and skeletal muscle mitochondria feature specialized structures called nanotunnels that allow component mixing between mitochondria which resemble a fusion event (Huang et al., 2013). Disruption of MtDy has drastic consequences for cell physiology which are mainly associated with the accumulation of dysfunctional and ROS-generating mitochondria and heart failure. In humans, mutations in MFN2 and OPA1 cause Charcot–Marie–Tooth disease type 2A and dominant optic atrophy respectively. Also, complete loss of mitochondrial fusion results in a dramatic decrease in mtDNA content, loss of membrane potential and reduced respiratory chain function in both cultured cells and tissues. After conditionally knocking out Mfn1 and Mfn2 genes in adult rats, the mitochondrial fission in cardiomyocytes increased leading to abnormal cellular respiration, eventually leading to progressive dilated cardiomyopathy. Excessive mitochondrial fission has been associated with decreased mitochondrial function and increased ROS (Jheng et al., 2011; Haileselassie et al., 2019). FIS1 up-regulation decreased cellular ATP levels in anoxic cardiomyocytes (Wang et al., 2012). On the other hand, an excessive fusion is also deleterious for cardiac cells. MFF mutant mice died at 13 weeks due to heart failure caused by severe dilated cardiomyopathy. Mutant tissues showed decreased mitochondrial density and respiratory chain activity, and increased mitochondria size (García-Palmer, 2008; Zhou et al., 2017; Zhou and Tian, 2018). A thoughtful review on mitochondrial dynamics and cardiac biology is found in Vasquez-Trincado et al. (2016).

Although the role and the importance of the proper balance of MtDy for cell physiology are well accepted, there is no clarity about the signaling mechanisms that might be associated with heart failure. In our laboratory studying the role of MtDy in erythropoiesis, it was discovered a link between the mPTP and MtDy to control cell differentiation in erythropoiesis (Gonzalez-Ibanez et al., 2020). Interestingly, the role of the mPTP in cellular differentiation has been reported in the past for early embryonic cardiomyocytes, where a frequent opening of the mPTP is needed to maintain an immature mitochondrial morphology with low oxidative capacity. On the other hand, the closure of the pore was required for proper differentiation into cardiac muscle cells (Hom et al., 2011; Folmes et al., 2012). One of the triggers of mPTP opening is an elevated Ca++ concentration in the mitochondrial matrix (Bernardi and Di Lisa, 2015) and the up-regulation of FIS-1 by means of its interaction with the ER protein BAP31 might favor calcium entrance. In fact, mitochondrial calcium overload is a key determinant in heart failure (Iwasawa et al., 2011; Santulli et al., 2015).

Mitophagy is the cellular process to dismiss dysfunctional mitochondria and secure ATP demand for heart cells. As it was discussed before, many pathways and proteins are implicated in mitophagy which responds to different stimuli like ROS, hypoxia and mitochondrial depolarization. The great variability and diversity of proteins and pathways controlling mitochondria elimination reflect the importance and complexity of mitophagy for cell function that we are still far to fully understand. In cardiac cells, it is clear that mitophagy plays a major role in keeping healthy mitochondria and many or all known mitophagy pathways have been shown to play a critical role. In fact, it was recently published a novel and astonishing mechanism in cardiac cells to get rid of dysfunctional mitochondria, especially under cardiac stress. Dysfunctional mitochondria are expelled out from cardiomyocytes in special vesicles called exophers which are made by means of the cardiac mitophagic machinery. These expelled mitochondria are engulfed by macrophages which are resident in the cardiac tissue. Blocking the mitophagy machinery, reduction of macrophages or ablation of the specific exopher receptor caused and accumulation of damaged mitochondria and dysfunction in the heart (Nicolás-Ávila et al., 2020). In general, disruption of mitophagy is associated with heart failure and cardiopathies Deep details on mitophagy and cardiovascular diseases can be found in Morciano et al. (2020). On the other hand, induction of mitophagy, by any means (exercise, food, specific drugs or hypothermia), has shown a protective effect on many cardiovascular diseases.

## Cardiac Mitochondria in Aging

Much of the cardiac literature describes a relationship between different cardiopathies and mitochondrial dysfunction. Daghistani et al. (2018), Chaanine (2019) showed that mitochondria changed their morphology from elongated to fragmented in heart failures. Lin and Kerkelä (2020), that there is a reduction in ETC and bioenergetic capacity in cardiomyopathies; and Vasquez-Trincado et al. (2016), Bravo-San Pedro et al. (2017) that mitochondrial dynamics and mitophagy are altered or reduced in cardiovascular diseases. In the end, most of those pathologies are related to an exacerbated amount of ROS, dysregulation of calcium homeostasis and the aperture of the mPTP. Mitochondria are constantly adapting bioenergetics, metabolism and signaling to allow cell function and survival. Thus, aged mitochondria (Table 1), with altered ROS and calcium homeostasis and dysregulation of the mPTP leading to ATP crisis, are one of the main causes of heart failure (Brand et al., 2013). Recently, the ANT protein was shown to increase mitochondrial proton leak - mitoflashes - in aged cardiomyocytes. This pathological proton leak is associated with inefficient ATP synthesis and increased electron flux and oxygen consumption, increasing ROS production and mPTP sensitivity. Interestingly, the treatment with the drug SS-31, a tetrapeptide that binds to cardiolipin-containing membranes and improves membrane stability and cristae curvatures (Birk et al., 2014), arrested proton leak, restored mitochondrial functionality, reduced ROS production and more surprisingly rejuvenated old cardiomyocytes (Zhang et al., 2020). Cardiac aging is characterized by a decrease in the number of mitochondria in the heart and a reduction in the area of the inner mitochondrial membrane implicating OXPHOS at the center of cardiac mitochondrial metabolism deficiency during aging. Respiratory complex III and IV deficiency in aging caused a decreased efficiency of OXPHOS, affecting also the number and mass of interfibrillar myofibrils (Stoll et al., 2018; Lin and Kerkelä, 2020).

The main driver of mitochondrial dysfunction in aging is the mtDNA, which progressively accumulates mutations and reduces its copy number as humans are getting old. In fact, mtDNA mutations cause aging, heart failure and many other aging-associated diseases. In 2004, it was published in Nature journal the “mutator mouse” whose mitochondrial DNA polymerase (POLG) had a dysfunctional proofreading domain which introduced random point mutations in the mtDNA, increasing mtDNA heteroplasmy (Trifunovic et al., 2004). The “mutator mouse” at the early age of 6 months, presented all the symptoms of old age such as blindness, deafness, alopecia, muscular atrophy, anemia, hump development and heart hypertrophy. The creation of this mutator mouse is an irrefutable proof that mtDNA mutations are a cause of aging and cardiovascular diseases. mtDNA mutations lead to OXPHOS inefficiency and then energy shortages especially in the tissues with the highest energy demands such as the heart, muscle and brain, which are the most vulnerable. By 40 weeks of age, mutator mice presented an enlarged volume of the left ventricle along with an increase in weight in relation to body weight. Histochemical analysis of the mutator mouse’s heart revealed a mosaic pattern with cytochrome c oxidase deficiency in some cardiomyocytes, which also occurs in the aged human hearts. TEM analysis of mitochondria ultrastructure confirmed the accumulation of fragmented, abnormal mitochondria. In addition, a decreased ATP production rate was noted (Trifunovic et al., 2004).

Another form of mtDNA damage during aging in humans is large mtDNA deletions. Mitochondrial diseases are usually associated with 5kb deletions of mtDNA which have been shown to accumulate in the human’s brain, muscle and heart (Chocron et al., 2019). Mutant mtDNA co-exists with wild type mtDNA giving rise to mtDNA heteroplasmy affecting the proper function of OXPHOS in a vicious cycle. Some pathogenic mtDNA mutations and a reduction in their copy number in cardiac cells have been specifically associated with impaired cardiac function and cardiac diseases and then, mtDNA mutations and copy number can be used as potential biomarkers of heart diseases (Ashar et al., 2017; Bray and Ballinger, 2017; Elosua, 2018; Galera-Monge et al., 2019). In fact, Elosua (2018) is emphatic when claiming that “mtDNA has been largely forgotten in cardiovascular research.”

The increase in mtDNA heteroplasmy not only affects mitochondrial function but also affects and influences nuclear genome mutations and the severity of cardiovascular diseases. This is also proof of mitochondria acting as a powerful signaling platform regulating both transcriptional and epigenetic nuclear gene expression. This mitochondria-nucleus retrograde communication acts in a synergistic way to produce cardiomyopathies or increase their severity. McManus et al. (2019) showed that ablation of ANT (the mitochondrial Adenine Nucleotide Transporter) in nuclear DNA is associated with cardiopathies where OXPHOS, MtDy and mitochondrial morphology are affected. However, in the presence of mtDNA mutations, cardiopathies are much more severe. mtDNA-related mitochondrial genetic diseases are associated with the development of cardiomyopathies in 40% approximately (Bates et al., 2012). Along with mtDNA mutations, the mtDNA haplogroups are also predictors of both lifespan and risk of several age diseases. Dr. Wallace’s group showed the type of mitochondrial haplogroups influences the severity of cardiovascular diseases with haplogroups U and H being the most affected (Strauss et al., 2013). On the other hand, the haplogroups J and T are associated with a reduced risk of cardiovascular disease (Veronese et al., 2019).

Reduction in heteroplasmy and restoration of mtDNA copy number is achieved by the coupling of OXPHOS with the Mitochondrial Life Cycle. It has been reported damaged DNA invokes mitophagy (Dan et al., 2020) and that adult post-mitotic tissue can eliminate mitochondria carrying damaged mtDNA by mitophagy which help to preserve cellular and mitochondrial function, suggesting that removal of the mutant mtDNA is protective for cells (Kandul et al., 2016). Mitochondrial turnover decreases with age, so there is growing interest in enhancing the pathways of mitophagy by discovering new pharmacological targets or cell mechanisms to counteract mitochondrial heteroplasmy (Diot et al., 2016). The aggravation of cardiac aging together with the accumulation of damaged mitochondria in cells can be caused by impaired and deficient mitophagy that results in increased heteroplasmy and altered mitochondrial metabolism (Quan et al., 2020). In fact, many of the pathways that improve health and extend longevity in various organisms all converge on mitophagy which is dependent on mitochondrial biogenesis and dynamics. A decrease in mitochondrial fission, functional mitochondria and NIX and FUNDC-1 receptor-mediated mitophagy may result from mtDNA mutations associated with aging (Lampert et al., 2019; Wu et al., 2019). Furthermore, Woodall et al. (2019) examined the role and expression levels of Parkin on accelerated cardiac failure in the mtDNA POLG mutator mice. It was observed that Parkin decreases in the mutator mouse’s heart with age. However, the restoration of Parkin level by means of its overexpression did not rescue the cardiac hypertrophy in the POLG mouse. Besides, deletion of parkin did not worsen heart disease. The authors claimed that mitochondria were not altered in their bioenergetics and that their function did not decline with age. This author’s interpretation is controversial since they observed a clear and significant decrease in complex II and IV protein levels, and TEM images showed altered cristae morphology in mutant mice with age, which might be indicative of mitochondrial dysfunction. An interesting discovery in the POLG mutant mouse’s heart is the upregulation of the receptor-mediated mitophagy genes and the appearance of larger mitochondria, which is in agreement with the upregulation of NIX protein myocardial hypertrophy (Yussman et al., 2002). This larger mitochondrial phenotype might be due, among other causes, to a decreased production and expulsion of exospheres that are dependent on the autophagic machinery (Nicolás-Ávila et al., 2020). However, the reduction of parkin in aging may be compensated by the upregulation of MUL1, a ubiquitin E3 ligase that is involved in regulating mitochondrial dynamics by promoting MFN2 degradation and leading to mitochondrial fragmentation and Parkin independent-mitophagy (Yun et al., 2014; Cai and Jeong, 2020). In cardiac cells, upregulation of MUL1 has been shown to fragment mitochondria and alter cristae structure, reduce mitochondrial membrane potential, promote cytochrome c release and sensitize mPTP, making it a potential mechanism of cell death and heart failure in aging (Yang et al., 2016; Wang et al., 2020).

Stressed mitochondria have been shown to release mtDNA into cytosol which is recognized as a Damage-Associated Mitochondrial Patterns (DAMPs), that trigger an inflammatory response. This pro-inflammatory environment may activate fibroblast proliferation and excessive production of extracellular matrix proteins which correlates with cardiomyopathies (West et al., 2011; Lin and Kerkelä, 2020). These alterations at molecular levels are associated with cellular changes such as hypertrophy, fibrosis, accumulation of misfolded proteins, loss of cardiac cells, extracellular matrix remodeling and amyloid deposition and structural changes in the myocardium like left ventricle hypertrophy and left auricle hypertrophy, which causes diastolic dysfunction (Steenman and Lande, 2017; Liang and Gustafsson, 2020). It has also been reported that mtDNA and whole mitochondria are released into bloodstream which offers a new biomarker of mitochondrial function and stress. mtDNA may travel naked (circulating cell-free mtDNA) which is recognized by immune cells to initiate the immune response, or they can go inside extracellular vesicles (EVs). Circulating EVs have been described as a novel mechanism of intercellular communication. In aging, Lazo et al. (2020) showed that mtDNA in EVs is diminished as compared with young people and even more, EVs containing old and damaged mtDNA affects mitochondrial bioenergetic in other cells and tissues. Picca et al. (2019) pointed out that EVs allow the elimination of dysfunctional or damage mitochondrial components; and Freeman et al. (2018) proved that EV secretion is dependent on autophagy. All these antecedents support the hypothesis that a decline in mtDNA containing EVs is due to a defect in mitophagy with aging.

## Linking OXPHOS, Mitochondrial Life Cycle and mtDNA: Signaling Pathways

The master regulator of mitochondrial biogenesis is the protein PGC-1α which is a transcriptional co-activator that induces nuclear- and mitochondrial- encoded gene transcription coordinating both genomes. PGC-1α can be stimulated/induced by physical exercise, cold and hypoxia. The signaling pathways to activate PGC-1α are diverse and well described (see Gureev, 2019; Oka et al., 2020). In mammals, an increase in the AMP/ATP ratio given by an overwhelming ATP demand or reduction in ATP synthesis activates AMPK (AMP protein kinase) which in turn will activate PGC-1α and inhibit mTOR (mechanistic target of rapamycin kinase). In this way, AMPK activation stimulates mitochondrial biogenesis and mitophagy (Kupr and Handschin, 2015). As a transcriptional co-activator, PGC-1α does not bind directly to DNA promoters but interacts and activates the transcription factors Nuclear Respiratory Factor 1 and 2 (NRF1 and NRF2), Estrogen-Related Receptor (ERR) and Peroxisome Proliferator Activated Receptor (PPAR). In general, NRF2 induces nuclear-encoded OXPHOS protein expression and NRF1 induces the expression of mitochondrial transcription factors TFAM and TFB to initiate mtDNA replication and transcription. ERR and PPAR are involved in mitochondrial biogenesis and dynamics, Krebs’ cycle and mitochondrial fatty acid oxidation expression genes. Furthermore, the increase in NAD + /NADH ratio as a result of the electron transport chain induces SIRT1 that also stimulates PGC-1α.

An essential component in mitochondrial signaling is ROS. In addition, ROS regulates mitochondrial dynamics by promoting mitochondrial fission and disrupting mitochondrial fusion, decreasing the OPA1 active isoform and degrading MFN1/2; and mitophagy. In cardiac myocytes, increased ROS induces a non-canonical function of the human Telomerase Reverse Transcriptase (hTERT), which reversibly translocate from the nucleus to the mitochondria to bind and protect mtDNA, reduce ROS production and increase OXPHOS efficiency (Haendeler et al., 2009; Ait-Aissa et al., 2019). Besides, the up-regulation of TERT is dependent on PGC-1α (Zhang et al., 2018) and positively regulates PINK1 function and stabilizes its mitochondrial localization to promote mitophagy (Shin and Chung, 2020). PINK1 other than phosphorylating ubiquitin and Parkin to initiate mitophagy, phosphorylates PARIS and leads to its Parkin ubiquitination and elimination. Therefore, when the PINK/Parkin axis is inhibited, PARIS accumulates and represses PGC-1α (Lee et al., 2017); and the knockdown of PARIS with CRISPR/Cas9 regains mitochondrial biogenesis (Kumar et al., 2020).

There is another parallel and independent pathway in controlling mitochondrial biogenesis given by the transcription factor Nuclear Erythroid-Related Factor 2 (NFE2L2. ^∗^It is also named NRF2, but in this work will be called NFE2L2 to avoid confusion). It has been described that NFE2L2 can translocate to the nucleus under an increase in ROS, specifically H2O2 and binds to the antioxidant response elements (ARE) in the promotor of NRF1 to induce TFAM. In nematodes, it was found that the orthologous protein of human NIX/BNIP3, named DCT-1 that works together with PINK and PRD (Parkin orthologous) is controlled by SKN-1, which is also linked to mtDNA replication. SKN-1 deficient nematodes display reduced mitophagy and reduced mitochondrial biogenesis. Even stimulation of mitophagy does not work in SKN-1 KO cells (Palikaras et al., 2015b). SKN-1 is a sensor of oxidative stress generated by defective mitochondria and is required for the expression of several genes related to mitochondrial biogenesis. SKN-1 is the orthologous of human NFE2L2 (Palikaras et al., 2015b). In mammals, Ivankovic et al. (2016) established the relationship among mitochondrial damage, NFE2L2 induction, P62, mitochondrial biogenesis (mtDNA replication) and protection against oxidative stress, in addition to lysosomal biogenesis to support the formation and degradation of autolysosomes. Increased autophagy involves an interaction between the autophagy adaptor p62/SQSTM1 and KEAP1, the cytosolic inhibitor of NFE2L2 allowing the accumulation of NFE2L2 in the nucleus, and then increased expression of nuclear-encoded mitochondrial genes needed for mitochondrial biogenesis and antioxidant response (Ichimura et al., 2013; Katsuragi et al., 2016).

In aging, it has been widely reported a decreased mitophagy (Wu et al., 2019; Bakula and Scheibye-Knudsen, 2020) commanded by a reduction of Parkin protein expression and the concomitant upregulation of the Nuclear Receptor Corepressor 1 (NCoR1) and its homologous protein SMRT, which counteracts the transcriptional control commanded by the SIRT1-AMPK/PGC1-alpha axis. In addition, NCoR1 will downregulate lipogenic and antioxidant genes favoring the metabolic switch from lipid oxidation to glucose oxidation and diminishing the antioxidant capacity of cells. NCoR1 KO animal models have greater mitochondrial biogenesis, mitophagy and lifespan (Perez-Schindler et al., 2012; Fan et al., 2013; Liu et al., 2013; Ou-Yang et al., 2018). The stabilization of NCoR1 in aging is because this corepressor is normally bound to LC3 family protein and degraded by mitophagy. Arrested mitophagy will cause its accumulation and downstream effects (Saito et al., 2019). Despite these antecedents, the role of NCoR1 in cardiac aging is still controversial and needs more research. Li et al. (2019a) suggested that NCoR1 has a protective cardiac effect by suppressing cardiac hypertrophy and Wang et al. (2018) have shown that NCoR1 is downregulated in old mouse hearts. In this regard, NCoR1 is not the only transcriptional co-repressor involved in aging but also there is SMRT and RIP140 that might play a relevant role (Fan et al., 2013; Liu et al., 2013).

## Proposed Model of Cardiac Aging

**FIGURE 2:**
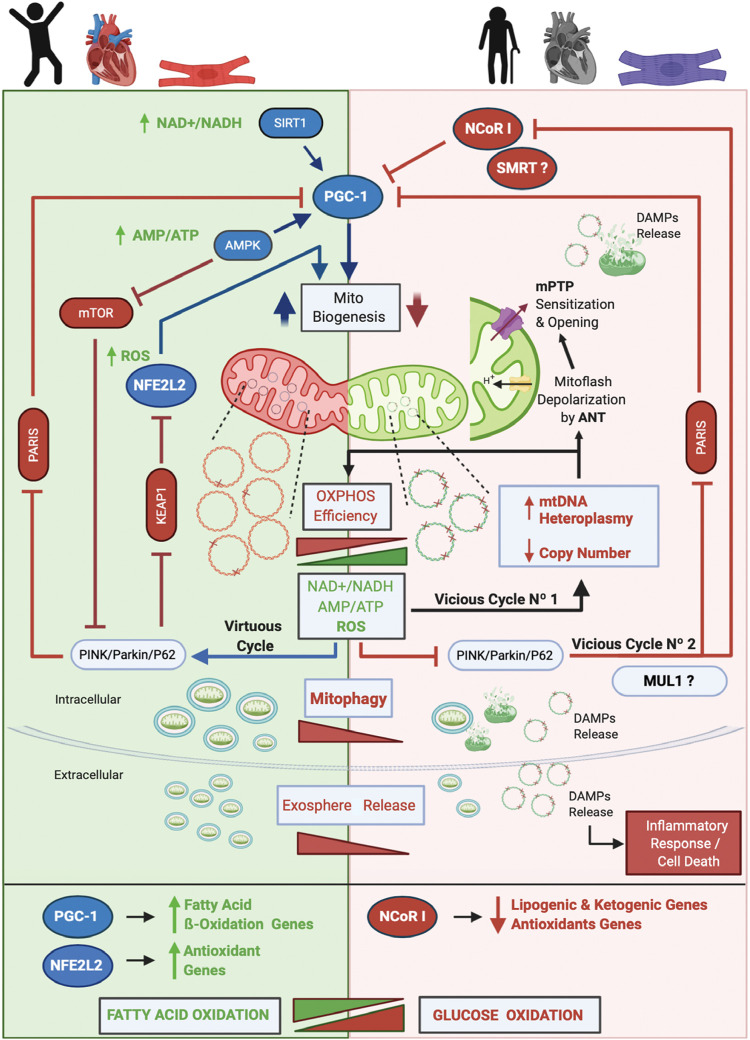
Proposed Model of Cardiac Aging. The core of our model and the main driver of aging is mtDNA heteroplasmy. In aging, two vicious cycles were defined. Vicious cycle No1 will progressively increase the level of mtDNA heteroplasmy and decrease OXPHOS efficiency at the point to affect mitophagy and set the onset of Vicious cycle No2 that will progressively inhibit mitochondrial biogenesis and antioxidant response due to inhibition of PGC-1α and stimulation and upregulation of NCoR1. As a result, dysfunctional mitochondria will accumulate, increasing the number of mitoflashes and pathological ROS to finally trigger the mPTP opening and the release of DAMPs to initiate an inflammatory cell response and an apoptotic/necrotic stimulus. Furthermore, a metabolic switch from fatty acid oxidation to glucose oxidation will occur. Altogether, these events lead to cardiovascular diseases and cardiac failure. On the other hand, in young people, a low level of mtDNA heteroplasmy will keep the efficiency of the OXPHOS system which will maintain low NAD + /NADH and AMP/ATP ratios and a physiological amount of ROS. These conditions allow the Virtuous Cycle where MtDy will segregate dysfunctional mitochondrial units for degradation by mitophagy and will activate PGC-1α and NFE2L2 for mitochondrial biogenesis. Of note, cardiac cells are able to expel out mitochondria in vesicles called exospheres that are degraded by surrounding macrophages. In this model, we hypothesized that exosphere release, which is dependent on the autophagic machinery, is decreased in aging. Also, it is not clear yet whether in cardiac aging the protein MUL1 is involved in mitophagy, but it has been shown to be upregulated under cardiac damage. Along with NCoR1, another transcriptional corepressor like SMRT has been shown to be involved in aging.

The core of our model and the main driver of aging is mtDNA heteroplasmy (Figure 2). In young people with a healthy heart and cardiomyocytes, OXPHOS efficiency is high and the mtDNA mutation level, is low (mutated mtDNA is represented with one or more “X” on the double-stranded circular DNA). Under this scenario operates the mitochondrial life cycle to dilute mutated copies of mtDNA by means of MtDy and mitophagy. This is what it is called in our model The Virtuous Cycle. One of the main controllers of mitophagy is the PINK/PARKIN/P62 axis. When active, two repressors of mitochondrial biogenesis, PARIS and KEAP1 are inhibited and degraded, unleashing the transcriptional coactivator PGC-1α and the transcription factor NFE2L2 which are also activated by AMPK -via increased AMP/ATP ratio- and ROS respectively. AMPK will also inhibit the mTOR pathway to induce mitophagy. In addition, increased NAD + /NADH activates SIRT1 which also induces PGC-1α for mitochondrial biogenesis. Once the autophagosomes are formed, they are eliminated via their fusion with lysosomes or expelled out of the cell as exospheres, to be engulfed by macrophages. The latter is a new mechanism described only for cardiomyocytes.

As mtDNA heteroplasmy exceeds 60%, mtDNA replication and transcription will be affected, reducing mtDNA copy number and proteins needed for respiratory chain assembly, leading to mitochondrial dysfunction and disruption of mitophagy. As a result, dysfunctional mitochondria will accumulate within cells, and cellular bioenergetics needs will not be satisfied causing an ATP-crisis, cell atrophy or death, ultimately leading to aging and aging-associated diseases. In fact, aging-associated diseases such as cancer, diabetes, heart disease, muscle weakness or atrophy, Alzheimer’s disease and Parkinson’s disease among others, are mainly due to a loss of mtDNA integrity and a failure in the mitochondrial life cycle (biogenesis, MtDy, mitophagy). To date, more than 400 mtDNA mutations, as well as a reduction in mtDNA copy number and a decrease in mitophagy, have been associated with human diseases (Tuppen et al., 2010; Li et al., 2019b). mtDNA mutations and heteroplasmy will affect the performance of the OXPHOS system reducing its efficiency due to the progressive loss of respiratory complex assembly and coupling, generating an abnormal amount of ROS. This ROS will produce more mtDNA mutations, increasing progressively the level of mtDNA heteroplasmy. This process corresponds to the Vicious Cycle No1 in our model. ROS not only affect mtDNA but also proteins and lipids. Thus, the mtDNA replication machinery, as well as membrane proteins and membrane phospholipids, including cardiolipin will be damaged. In this regard, ANT protein has been associated with increased mitoflash generation (transient mitochondrial depolarization), which uncouples ATP synthesis from the electron transport chain and ROS generation. Under these conditions, the mPTP is prone to open, collapse mitochondrial function, release mtDNA and other DAMPs, and trigger an inflammatory response and cell death. In parallel, an excessive amount of ROS inhibits the PINK/PARKIN/P62 axis meaning that PARIS and KEAP1 will be active and then NFE2L2 and PGC-1α inactive. Then, mitochondrial biogenesis will be reduced. In addition, inhibition of the PINK/PARKIN/P62 axis will activate NCoR1 which in turn blocks PGC-1α and mitochondrial biogenesis. This is called the vicious cycle No2. As mitophagy gets decreased, it is expected that exosphere release is also affected, accumulating in the cytosol many dysfunctional mitochondria prone to release more DAMPs and to induce cell death. Another important feature, in aging, is the metabolic switch from fatty acid oxidation to glucose oxidation, given the inhibition of NFE2L2 and PGC-1α which upregulates the fatty acid oxidation and antioxidant genes; and the activation of NCoR1 which down-regulates the lipogenic and ketogenic genes, and also the antioxidant genes. All these metabolic changes occurring in aging find their onset in the heteroplasmic mtDNA; and the final consequence of this, is having dysfunctional and hypertrophic cardiomyocytes leading to heart failure.

## Mitochondrial Therapies for a Healthy Heart

Decreased heteroplasmy, increased mtDNA copy number, increased mitochondrial biogenesis and mitophagy have been shown to cause rejuvenation. Old mice that had a substantial loss of mtDNA copy number and reduced mtDNA gene expression showed improved memory performance after mtDNA replication and transcription were stimulated. The transgenic mouse called “mtDNA depleter” evidenced skin wrinkles and hair loss, associated with reduced copies of mtDNA. However, by restoring mtDNA copies, the same animal rejuvenated and returned to normal. In transgenic mice expressing PGC-1α under the MKC promoter, overexpression of PGC-1α promoted mitochondrial biogenesis in skeletal and cardiac muscle from fetal life onward, ameliorated aging phenotypes and prevented aging-associated cardiomyopathies in adults (Yuan et al., 2016). Regarding mitophagy, pharmaceutic compounds, calorie restriction or genetic manipulation have been proven to augment lifespan in different organisms via increased mitophagy. Upregulation of systemic ATG5 expression, a protein involved in autophagosome formation, in transgenic mice have increased mitophagy resulting in improved health, longer life spans and less weight gain with aging (Pyo et al., 2019). Besides, enhanced mitophagy and less fibrosis during aging were found in those mouse hearts. This was also established by the Levine group and others that constitutive activation of the autophagic gene Beclin1 enhanced autophagy resulting in remarkable health improvement and a longer lifespan in mice. Additionally, those mutant mice when aged, have less interstitial fibrosis and cardiac hypertrophy, meaning that maintenance of autophagy in the heart postpones or avoids cardiac aging and protects the heart during sepsis (Fernández et al., 2018; Sun et al., 2018). Even in injured cardiac tissue, mitophagy stimulation improves cellular function via a decrease in ROS and apoptosis, and mitochondrial function improvement (Torrealba et al., 2017). After ischemia/reperfusion injured heart, hypothermia has been shown to protect the cardiac tissues by increasing autophagy, autophagy flux and mitochondrial content in farm pigs (Marek-Iannucci et al., 2019). On the other hand, autophagy and autophagic flux impairment in the heart caused its accelerated aging, remodeling it toward heart hypertrophy and dysfunctional mitochondria, which are associated with heart failure and cardiomyocyte degeneration (Takemura et al., 2018; Liang and Gustafsson, 2020).

In the biomedical field, there is a growing interest in enhancing mitochondrial function to combat aging-associated diseases and improve lifespan. This has led to the development of nutraceutical therapies based on the application of antioxidants, vitamins or respiratory substrates and the discovery of new drugs, which have shown a positive antiaging effect. Some of the well-known compounds are resveratrol, an antioxidant found in red fruits and red wines; and rapamycin, a macrolide compound that is a blocker of the mTOR pathway, and then a powerful inducer of mitophagy, prolonging life in mice and other model organisms and it prevents age-associated symptoms in mammals including humans. The ketogenic diets (low glucose, high ketone bodies), albeit their mechanism of action is not well understood, have been shown to inhibit mTOR, stimulate mitophagy, mitochondrial biogenesis and mtDNA replication, and reduce oxidative stress (Santra et al., 2004; Wallace et al., 2010; Gano et al., 2014). The novel discoveries, drugs or technologies include the catechinic acid, a polyphenol widely present in tea and fruits, that is able to induce mitophagy, improve fitness and extend lifespan in aged nematodes (*C. elegans*) (Wu et al., 2020); the catalpol, an iridoid glucoside widely abundant in the root of *Rehmannia glutinosa*, that has a powerful antioxidant effect, decreases DNA damage and stimulates the PGC-1α TERT axis (Zhang et al., 2018). The Telomerase Activator 65 (TA-65), a compound extracted from *Astragalus membranaceus* has been also shown to extend lifespan by increasing TERT expression and reducing markers of cardiovascular disease and inflammation (Fernandez et al., 2018). The tetrapeptide SS-31 binds to cardiolipin from the inner mitochondrial membrane, stabilizing membranes and the integral membrane mitochondrial proteins, in particular, ANT protein to prevent and even revert aging-associated mitochondrial dysfunction in cardiomyocytes (Szeto, 2006; Birk et al., 2014; Zhang et al., 2020). Finally, the thiazolidinedione pioglitazone has been shown to be a potent inductor of the PGC-1α, mitochondrial biogenesis and antioxidant mitochondrial defense (Bogacka et al., 2005; Butterick et al., 2016).

The correlation between mtDNA integrity loss and aging has allowed the development of experimental strategies that seek to eliminate mutated mtDNA or increase wild type mtDNA to restore mitochondrial function and reverse the aging phenotype. The rationale of these strategies is that, if the ratio between the wild type mtDNA and the mutated mtDNA can be adjusted, the presence of wild type molecules will mitigate the effect of the mutants and therefore the defect will be corrected. Four different strategies have been developed (Taylor and Turnbull, 2005; Patananan et al., 2016; Aravintha Siva et al., 2019),” seems to be incomplete. Kindly check and advise. (i) Transfer of isolated xenogeneic mitochondria, which are deposited in the cell culture medium and enter the cells by macropinocytosis (Kitani et al., 2014). It has recently been shown that mitochondria therapy is capable of promoting the regeneration of damaged hippocampal neurons (Katrangi et al., 2007; Chien et al., 2018; Kim et al., 2018). Other mitochondria transfer technique is by centrifugation and also by the generation of mitocytoplasts (Yang and Koob, 2012). (ii) Transfer of exogenous wild type mtDNA, by direct microinjection of mtDNA into the host cell or by the use of “carriers” such as the mitochondrial transduction domain (MTD) conjugated to the mitochondrial transcription factor A (MTD-TFAM), MITO Porters, DQAsomes, and nanoagents (Niazi et al., 2013; Zhang and Zhang, 2016; Aravintha Siva et al., 2019). Exogenous mtDNA has also been transferred into muscle cells via hydrodynamic limb venous injection (Yasuzaki et al., 2013). These methodologies have shown promising and surprising results in the rescue of mitochondrial and cellular function. (iii) Elimination of heteroplasmy. These techniques are named the antigenomic mtDNA Therapy; the mitochondrial-targeted restriction endonucleases; zinc-finger nucleases type; and the effector nucleases of the transcription activator type (Transcription Activator-like effector Endonucleases), are all based on the delivery of endonucleases into mitochondria, which will specifically recognize and degrade the mutated mtDNA. An attempt has also been made to carry out the CRISPR/Cas9 technology in mitochondria, but the results have not been reproducible (Jo et al., 2015). (iv) Mixed mtDNA transfer system. This technology proposes direct electroporation of isolated exogenous mitochondria with wild type mtDNA. Next, mitochondria must then be transferred to the zygotes or cultured cells through microinjections (Collombet et al., 1997; Yoon et al., 2010). Recently, it was published in Nature journal a new technology to edit mitochondrial DNA which combines mitotalen proteins, Cas9 and the Uracyl glycosidase inhibitor with a bacterial cytidine deaminase toxin. This technology acts as a repair/edit system on mutant forms of mtDNA. It does not eliminate mtDNA copies, but rather, restore them (Mok et al., 2020). This revolutionary system was tested in HEK293T cells and it remains open to the question if it will also be efficient in other cellular models like cardiac cells.

In summary, heart mitochondria must satisfy an enormous amount of energy to keep the heart beating from conception to death. To do that, mitochondria are equipped with two complex systems that work collaboratively, the OXPHOS system and the Mitochondrial Life Cycle to keep safe and healthy the mtDNA and then mitochondria. Any disruption of these processes is associated with heart failure. On the other hand, the potentiation of these is beneficial to improve heart health. In aging, mtDNA is prone to undergo mutations leading to heteroplasmy which is associated with increased ROS affecting negatively both OXPHOS and the mitochondrial life cycle. Mitochondrial therapies have been developed to deal with mtDNA heteroplasmy. Many of them focus on Mitophagy stimulation which decreases mtDNA heteroplasmy and has a protective effect in cardiac cells. The development of new therapies aims to decrease mtDNA heteroplasmy in cells either by the transfer of mutation-free wild type mtDNA or by the direct elimination of mutant mtDNA. The results of these new technologies are promising in restoring mitochondrial and cellular function *in vitro* and open a new era in the field of mitochondrial medicine to combat aging-associated heart failure.

## Author Contributions

JS helped with the writing and discussion of the manuscript. AE conceived, wrote, and funded the manuscript. Both authors contributed to the article and approved the submitted version.

## Conflict of Interest

The authors declare that the research was conducted in the absence of any commercial or financial relationships that could be construed as a potential conflict of interest.
